# Terminal sterilization of food-grade coatings for cellular agriculture: robustness against water quality and regulatory feasibility

**DOI:** 10.1016/j.btre.2026.e00976

**Published:** 2026-08-04

**Authors:** Hiroaki Hatano, Misaki Sawada, Naomi Nakamura, Misaki Kitsukawa, Azusa Hayakawa, Hiroaki Kondo, Ikko Kawashima

**Affiliations:** IntegriCulture Inc., Shonan Health Innovation Park (Shonan iPark), 2-26-1, Muraoka-Higashi, Fujisawa, Kanagawa, 251-8555, Japan

**Keywords:** Cellular agriculture, Food-grade cell culture, Regulatory science, Gelatin coating, Terminal sterilization

## Abstract

High production costs remain a major obstacle to the commercialization of cell-based foods, primarily due to the reliance on costly pharmaceutical-grade reagents and infrastructure. This study introduces a practical solution using a dual technical/regulatory approach. We developed iCoater, a food-grade coating agent manufactured via terminal heat sterilization, a standard in food processing. This process results in no detectable bacterial growth and 12-month stability without filtration. In performance tests using primary duck liver-derived cells, iCoater showed efficacy in cell attachment and proliferation. Furthermore, we confirmed that this performance and low endotoxin levels were maintained when the tested water sources covered a wide hardness range from soft (< 1.0 mg/L) to hard (1468 mg/L) water. By aligning the food-grade manufacturing process, we proposed the regulatory pathway under Japanese legislation, identifying "gelatin-processed food" as a realistic and transparent labeling strategy. This integrated approach provides a practical reference for future developments in cellular agriculture.

## Highlights

• Terminal heat sterilization enables low-cost cell culture coating production.
• Safety and performance confirmed even using hard water (1468 mg/L) sources.
• The product remained endotoxin-free without pharmaceutical-grade water purification.
• Gelatin retains cell adhesion properties after 121 °C retort sterilization.
• "Gelatin-processed food" labeling provides a viable regulatory pathway.

## Graphical abstract

**Figure:**
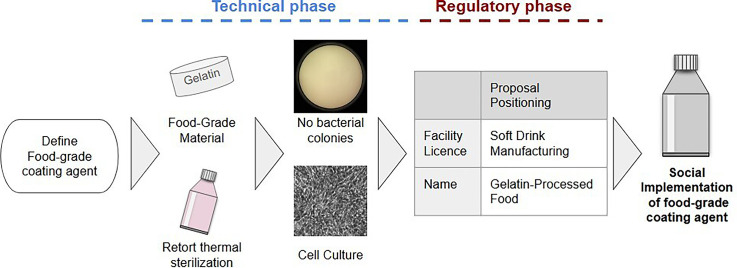

## 1 Introduction

Following marketing approval in Singapore and the United States, the social implementation of cell-based food is becoming a reality around the world (“ [[1], [2], [3], [4]]). However, progress in social implementation continues to be hindered by high production costs [[5], [6], [7], [8]]. To address this high-cost challenge, conversion into food-grade agents and processes is an effective strategy [[9], [10], [11], [12]]. In fact, the cost of pharmaceutical/research-grade materials and facilities required for cell culture can be hundreds to thousands of times higher than that of general food-grade equivalents [7,13]. This economic gap is one of the biggest barriers to the industrialization of cell-based foods.

Based on this idea, we focused on coating agents, which are the second most basic and versatile materials after culture media and are the foundation of adherent cell culture, which is in high demand. We selected food-grade gelatin primarily due to its global ubiquity as a common, widely available food ingredient, as well as its long history of use in food and low cost [[14], [15], [16]]. Indeed, a recent comprehensive review underscores that gelatin remains one of the most predominantly selected biomaterials for cultivated meat scaffolding due to its proven efficacy and accessibility [17]. Unlike complex growth factors, gelatin retains the Arg-Gly-Asp (RGD) sequences essential for integrin-mediated cell attachment even after thermal processing, as these peptide motifs are thermodynamically stable [18,19]. However, simply replacing the raw materials with cheaper food products will not bridge this economic gap. Because cell culture generally requires sterility, conventional cell culture materials have resolved the microbial risk through a process centered on expensive "sterilizing filters” [[7], [20]]. Because regular food production lines do not require the same standards as pharmaceuticals, including general viable bacterial counts, it is not easy to ensure sterility, which is a requirement for cell culture. As such, pharmaceutical approaches are optimized to meet the requirements of cell culture, which is essentially incompatible with inexpensive cell culture. Therefore, to achieve a true cost reduction, an inexpensive process that can guarantee sterility in food production lines is necessary as an alternative to sterile filtration. Furthermore, while cell culture typically requires pharmaceutical-grade ultrapure water (WFI), food manufacturing often utilizes potable or well water with varying hardness depending on the regional water source. Evaluating whether a cytocompatible, sterile coating can be consistently produced using such non-pharmaceutical water sources with varying hardness is highly meaningful for investigating the future techno-economic feasibility and global scalability of cellular agriculture. In this study, we focused on terminal sterilization, a well-established method in the food industry for products such as canned goods and retort pouches [21].

However, this technique presents new regulatory challenges. iCoater has a dual identity as both a ‘cell culture agent’ and a ‘food,’ driving ambiguity regarding its classification and labeling under existing food regulations [22,23]. Although the use of pharmaceutical-grade products is clear, when food manufacturing technology is applied, the definition of whether the final product is a ‘food’ or a ‘research reagent’ becomes unclear, and consistency with labeling regulations becomes a new issue. Currently, discussions focus on cell-based food itself [24], with a few examples focusing on culture agents. Countries are unlikely to make major changes to their existing regulations solely for nascent industries. Finding solutions within the current framework is the key to practical social implementation. Discussions on the establishment of rules regarding cell-based foods are underway, including in Japan [22,25]. To contribute to this discussion, commercial entities must first consider and present concrete options for classifying and labelling finished products under existing food regulations.

Thus, it is essential for social implementation to not only solve technical issues, but also simultaneously consider the regulatory path for social acceptance of technology. Building on our previous development of a food-grade cell dissociation agent [26], this study, using the food-grade coating agent ‘iCoater’ as a model case, we have integrated two themes: (1) technical issues (establishing the process to ensure sterility in general food production lines) and (2) regulatory discussions (the positioning of food-grade cell culture materials under current regulations), and presented a comprehensive solution for social implementation. The primary novelty and contribution of this study lie not in each individual component, such as the material selection, processing method, water robustness, or regulatory discussion, but in the validation of this integrated framework.

## 2 Materials and methods

### 2.1 Preparation for coating agent (iCoater)

The iCoater coating agent was prepared by replicating the food manufacturing process at the laboratory level. Food-grade porcine gelatin (Nippi Corporation, Tokyo, Japan AP-250F) was suspended in purified water at a final concentration of 0.1% (w/v) and sealed in a 2000 mL pressure resistant glass container (SCHOTT AG, Mainz, Germany; 3–743–0276) equipped with a high-temperature screw cap (GL-45) to ensure container-closure integrity. The suspension was then heated at 121 °C for 20 min using a high-pressure steam sterilizer (Hirayama, Saitama, Japan, HV-110ⅡLB) to simultaneously dissolve and sterilize the suspension (simulating the industrial retort sterilization process), providing a calculated *F_0_* value. To validate the sterilization efficiency, a spore challenge test was performed using a biological indicator (Attest 1262P; 3 M Company, St. Paul, MN, USA) containing *Geobacillus stearothermophilus* spores, confirming complete inactivation after the process. The suspension was cooled to room temperature and used as the product. The conventional coating agent used as a control was prepared by aseptically dissolving gelatin at the same concentration and sterilizing it by filtration using a 0.22 μm pore size filter (TPP, Rasadinge, Switzerland, 99,950).

To assess process robustness, iCoater was prepared using water samples with hardness levels ranging from soft water (hardness < 1.0 mg/L) to extremely hard water (1468 mg/L) (Table 1). The viscosity of the coating agent was measured by the Japan Food Research Laboratories (Tokyo, Japan).

**Table 1:** Robustness of iCoater manufacturing using specific tested water sources: Nominal quality and post-processing validation.

Water Source (Commercial Water)	Water Specifications (a) (Label-derived)		Measurement data (iCoater) (b) (Measured)			
	Hardness (mg/L)	pH	Endotoxin (EU/mL)	Sterility (CFU)	Mycoplasma	Cell Attachment (%) (c)
Purified Water (MilliQ water; Control)	< 1	7.0	<0.015	Not detected	Not detected	100± 6.5
Amidis (PT Amidis Tirta Mulia; Cimareme,Indonesia)	< 1	6.0–8.0	<0.015	Not detected	Not detected	112.4 ± 13.1
Kannon Onsen Nomu Onsen (Kannon Onsen Co., Ltd., Shizuoka, Japan)	< 1	9.5	<0.015	Not detected	Not detected	95.3 ± 10.4
Tsunan no Tennensui (Clear Water Tsunan Co., Ltd., Niigata, Japan)	17	7.0–7.5	<0.015	Not detected	Not detected	96.8 ± 5.8
Crystal Geyser (CG Roxane LLC, California, USA)	38	7.6	<0.015	Not detected	Not detected	82.7 ± 4.3
Nomu Onsen Veil (Shingenkan, Kanagawa, Japan)	40	9.8	<0.015	Not detected	Not detected	105.3 ± 7.6
Kirin Natural Mineral Water (Kirin Beverage Co., Ltd., Nagano, Japan)	53	6.9	<0.015	Not detected	Not detected	96.7 ± 4.6
Sarunage Onsen Kinsen no Mizu (Sanage Onsen, Aichi, Japan)	54.3	8.3	<0.015	Not detected	Not detected	95.7 ± 5.9
Kintaro no Chikaramizu (Minamiashigara City, Kanagawa, Japan)	86	7.6	<0.015	Not detected	Not detected	80.8 ± 6.2
Oishii Hadano no Mizu (Hadano City Waterworks Bureau, Kanagawa, Japan)	89	7.4	<0.015	Not detected	Not detected	111.7 ± 3.7
Acqua Panna (Sanpellegrino S.p.A., Tuscany, Italy)	107	7.1–7.9	<0.015	Not detected	Not detected	114.2 ± 13.6
Hakone no Mori Kara (Hakone Tozan Shoji Co., Ltd., Kanagawa, Japan)	120	7.4	<0.015	Not detected	Not detected	103.8 ± 10.6
HAVARY'S (HAVARY'S Inc., Saga, Japan)	190	7.5	<0.015	Not detected	Not detected	98.6 ± 3.7
Solan de Cabras (Mahou San Miguel, Cuenca, Spain)	260	7.74	<0.015	Not detected	Not detected	89.0 ± 2.5
Evian (Danone S.A., Auvergne-Rhône-Alpes, France)	304	7.2	<0.015	Not detected	Not detected	88.8 ± 6.8
Nanataki Onsen (YASURAGI group co., Ltd., Shizuoka, Japan)	416	8.5	<0.015	Not detected	Not detected	102.0 ± 5.1
Contrex (Nestlé Waters, Grand Est, France)	1468	7.4	<0.015	Not detected	Not detected	113.6 ± 3.1

Note: All validation parameters are valid strictly under the tested laboratory conditions.

(a) Values derived from the product labels.

(b) Validation data of the iCoater solution prepared with each water source.

(c) Relative cell attachment compared to the control (Purified Water). Data are expressed as mean ± SD (*n* = 3).

### 2.2 Sterility, endotoxin, and mycoplasma testing

To assess the sterility, the iCoater samples were plated on Soybean-Casein Digest (SCD) agar plates (Japan Becton Dickinson and Company, Tokyo, Japan, 252,479). After incubation, the number of colony-forming units (CFU) was determined using an automatic colony counter (Scan 500, Interscience, France).

The endotoxin concentration in the prepared iCoater samples was quantified using a kinetic limulus amebocyte lysate (LAL) assay kit (PYROGENT™ 5000, Lonza, Basel, Switzerland). The assay was performed strictly according to the manufacturer’s instructions.

Mycoplasma contamination was evaluated using two methods. For the iCoater reagent itself, the TaKaRa PCR Mycoplasma Detection Set (Takara Bio, Shiga, Japan) was used. To confirm the absence of mycoplasma in cells propagated using food-grade materials, genomic DNA was extracted using the Dneasy® Blood & Tissue Kit (Qiagen, Hilden, Germany), and PCR amplification was performed using the e-Myco™ plus Mycoplasma PCR Detection Kit (iNtRON Biotechnology, Gyeonggi-do, Korea). Amplification products were visualized using gel electrophoresis to confirm the absence of mycoplasma bands (all results were negative, as shown in Table 1).

### 2.3 Primary cell isolation from embryonic duck liver

Primary cells were isolated from embryonic duck liver. The fertilized duck eggs were incubated for 14 days in a P-008 incubator (Showa Furanki, Saitama, Japan) at 37 °C and 60% humidity for 14. For cell isolation, embryos were first washed with food-grade ethanol (Disinfectant Ethanol MIX; Kaneichi, Osaka, Japan) and I-MEM basal medium. The extracted liver tissue was dissociated into a single-cell suspension using a gentle MACS dissociator (Miltenyi Biotec, Bergisch Gladbach, Germany). The cell suspension was centrifuged at 300 × g for 4 min, and the pellet derived from two embryos was pooled and seeded into a single T175 flask to minimize individual variability.

### 2.4 Cell culture and performance evaluation

Before cell seeding, the culture plates were coated as follows. A sufficient volume of coating agent (iCoater or conventional reagent) was added to each well to completely cover the surface. The plates were then incubated at room temperature for 15 min. Following incubation, the excess solution was completely aspirated, and the cell suspension was immediately added to the wet surface without drying.

A heterogeneous population of primary cells was isolated from embryonic duck liver. Cells were maintained and cultured under standard conditions (37 °C, 5% CO_2_) in a food grade alternative basal medium, I-MEM (IntegriCulture, Tokyo, Japan) [10] supplemented with 10% Yolk Lipoprotein (YLP), a chicken egg-derived material used as a serum substitute [27,28], and 1% Penicillin-Streptomycin-Amphotericin B (PSA; Fuji-film-Wako, 161–23,181). For each passage, cells were detached using iDisper, a food-grade cell dissociation agent iDisper (IntegriCulture, Tokyo, Japan) [26], to maintain an entirely food-grade culture system. Two widely available commercial reagent-grade gelatin solutions were used as control coating agents: AscleStem® 0.1%-Gelatin Solution (Nacalai Tesque, Kyoto, Japan. 19,895–75; hereafter Reagent 1) and GLS250 Gelatin Solution (Nitta Gelatin; Osaka, Japan; hereafter Reagent 2).

Duck liver-derived cells were seeded at a density of 4 × 10^4^ cells/well onto 24-well plates precoated with either the developed product or a conventional coating agent. After 72 h of culture in I-MEM containing 10% YLP, the cells were detached, and the total cell number was determined using an automated cell counter. The cell engraftment and proliferation rates were calculated based on the total cell count after 72 h relative to the number of seeded cells.C2C12 and HEK293T cell lines were purchased from the RIKEN BioResource Research Center (BRC, Tsukuba, Japan). C2C12 cells were maintained in Dulbecco’s Modified Eagle Medium (DMEM; FUJIFILM Wako Pure Chemical, Tokyo, Japan, 043–30,085) supplemented with 10% fetal bovine serum (FBS; Thermo Fisher Scientific, MA, USA. A5256701). HEK293T cells were maintained in DMEM supplemented with 10% FBS. This setup was used to confirm that iCoater facilitates effective cell adhesion even when utilizing food-grade serum substitutes, consistent with the conditions used for primary duck liver-derived cells.

Primary duck liver-derived cells were seeded into white 96-well plates at a density of 2.0 × 10^4^ cells/well. After 24 h of incubation, cell attachment was quantified by measuring intracellular ATP levels using CellTiter-Glo® 2.0 Cell Viability Assay (G9241, Promega, WI, USA). Luminescence was measured using a Nivo multimode plate reader (PerkinElmer, MA, USA). The assay was performed strictly according to the manufacturer’s instructions. Relative attachment (%) was calculated by normalizing the luminescence values to those of the purified water control (set as 100%). To maintain consistency across evaluations, the luminescence at both 4 h and 72 h were normalized to the same purified water control, allowing the determination of relative cell attachment and proliferation.

### 2.5 Statistical analysis

All experimental data are expressed as the mean ± standard deviation (SD) from at least five biological replicates, unless otherwise noted. Statistical significance was determined using one-way analysis of variance (ANOVA), assuming normal data distribution. Multiple comparisons were performed using the Bonferroni post-hoc test. In all cases, a *p*-value of <0.05 was considered statistically significant. All analyses were performed using data from representative experiments, and similar results were confirmed in at least three independent experiments.

## 3 Results and discussion

### 3.1 Technical feasibility and biological performance

**Fig. 1:**
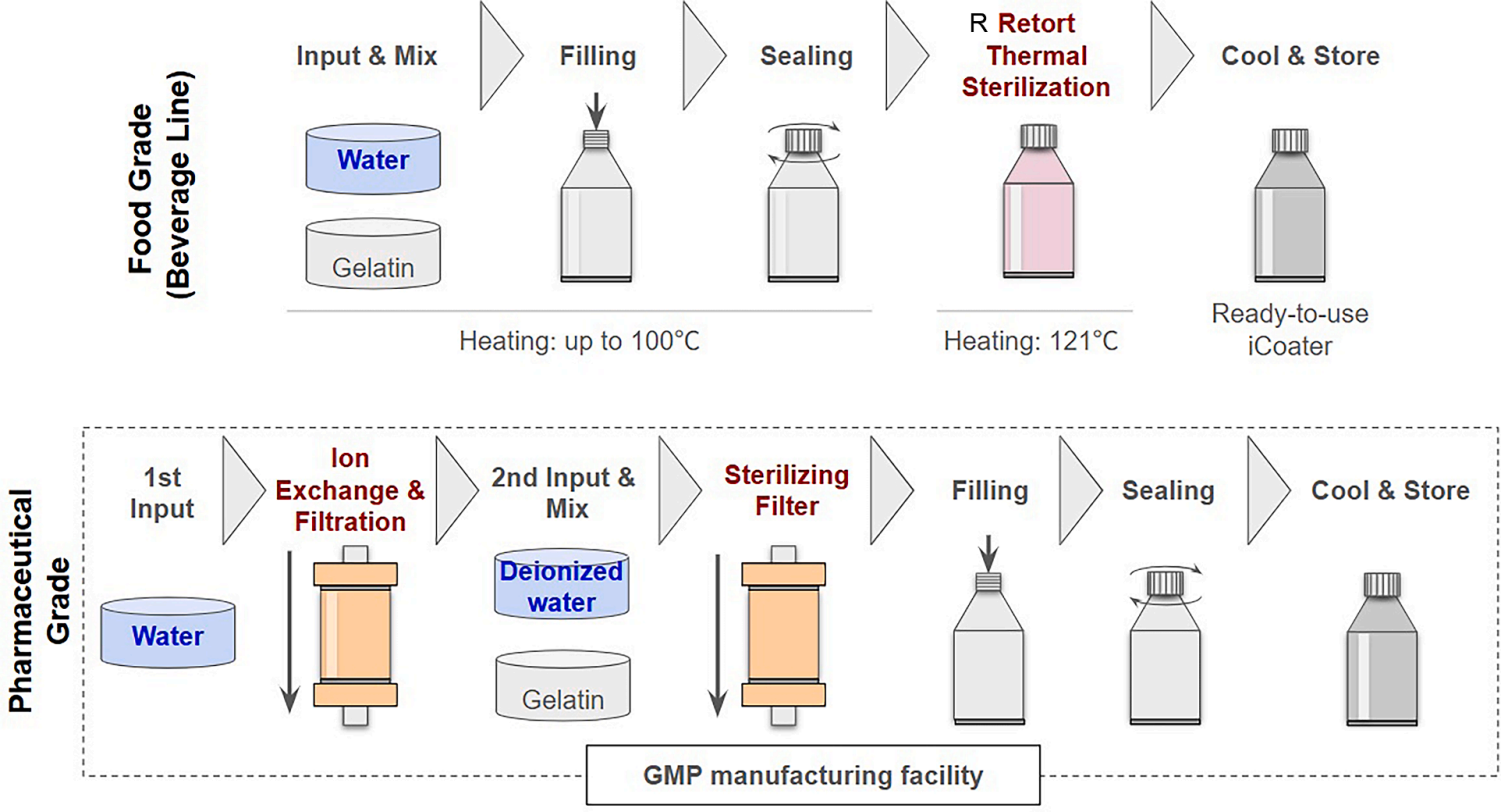
**Conceptual comparison of coating agent manufacturing processes.** The upper panel illustrates the developed food-grade process conducted in a Food Grade (Beverage Line), where dissolution and sterilization of 0.1% gelatin are integrated into a single Retort Thermal Sterilization (121 °C for 20 min) within a sealed container. The lower panel shows the Pharmaceutical Grade process in a GMP manufacturing facility, requiring Ion Exchange & Filtration and a separate 0.22 µm Sterilizing Filter before filling. Red labels indicate the corresponding sterilization and water purification steps between the two processes.

In this study, we established a manufacturing process for iCoater based on terminal sterilization, as compared to the conventional pharmaceutical-grade process in Fig. 1. In the Standard Food Factory (Beverage Line) model, the process achieves simultaneous dissolution and sterilization through Retort Thermal Sterilization (121 °C for 20 min). This contrasts with the Pharmaceutical Grade workflow in a GMP manufacturing facility, which necessitates two distinct stages of physical removal: Ion Exchange & Filtration for the water supply and a subsequent Sterilizing Filter for the product. By leveraging terminal sterilization, the iCoater process eliminates these filtration-dependent steps.

**Fig. 2:**
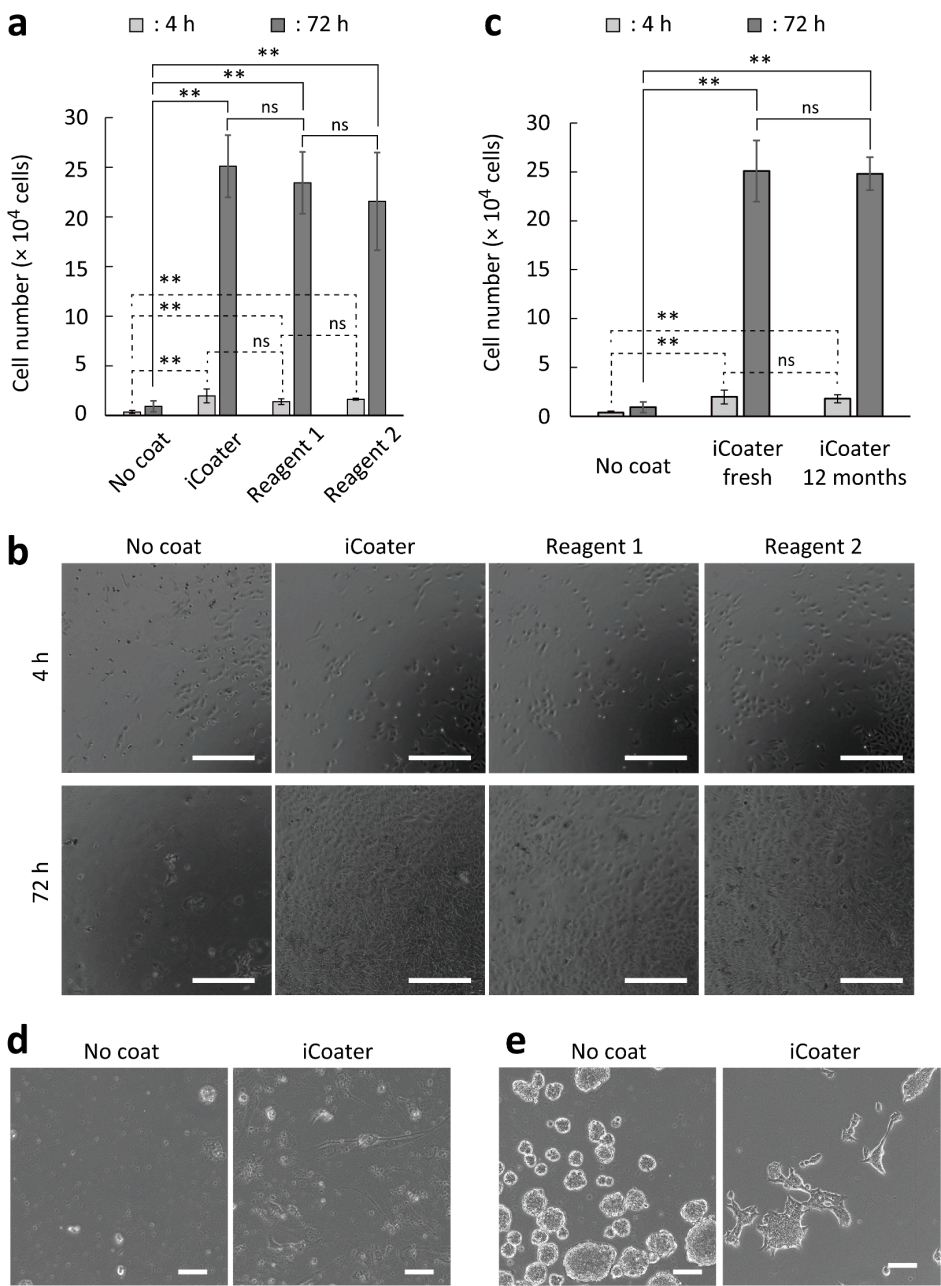
**Sterility and cell culture performance of iCoater.** (a) A comparison of duck liver-derived cell numbers at 4 and 72 h of cultivation on an uncoated surface (No coat) or surfaces coated with the iCoater, Reagent 1, or Reagent 2, with multiple comparisons performed at both the 4 h (dashed brackets) and 72 h (solid brackets) time points. Both initial cell attachment at 4 h and subsequent cell proliferation at 72 h were significantly enhanced on all coated surfaces compared to the uncoated control. Data are presented as the mean ± SD (*n* = 5; ** *p* < 0.01). ns: no significant difference (b) Bright-field microscopy images of the cells under each coating condition at 4 h and 72 h. Although cells on all coated surfaces reached confluence by 72 h, those on the uncoated surface did not. Scale bar = 400 µm. (c) Long-term stability of iCoater. Primary duck liver-derived cells were cultured for 4 and 72 h on plates coated with either freshly prepared iCoater (iCoater fresh: within 1 week) or iCoater that had been stored at 4 °C for 12 months, with multiple comparisons performed at both the 4 h (dashed brackets) and 72 h (solid brackets) time points. Data are presented as mean ± SD (*n* = 5; ** *p* < 0.01). ns: no significant difference (d) Morphological evaluation of C2C12 cells on Day 4 of culture with or without iCoater coating. (e) Morphological evaluation of HEK293T cells on Day 4 of culture with or without iCoater coating. Scale bars for (d) and (e) = 100 µm.

We verified that the iCoater manufactured using this process maintained its quality as a culture agent. CFU counting revealed no bacterial colonies, confirming the aseptic nature of the iCoater (Fig. 2a). The process produced samples with no detectable bacterial growth under the tested conditions (0 CFU across 69 independent replicate samples, *n* = 69). This reproducibly demonstrates that the terminal sterilization process reduced potential microbial risks under a calculated *F*_0_ value of 19.54. Additionally, the spore challenge test using a biological indicator containing *Geobacillus stearothermophilus* spores confirmed complete inactivation after the process, validating the robust sterilization efficiency (Figure S1). In the culture test, the primary duck liver-derived cell number and bright-field images after 72 h of seeding, indicating cell adhesion and proliferation, were not significantly different from those of conventional reagents (Reagents 1 and 2) (Fig. 2b). To evaluate cell culture, the proliferation rates were determined by calculating the fold increase in cell number from 4 h to 72 h, where the 4 h time point represents initial cell adhesion and the 72 h time point indicates subsequent cell proliferation. The fold increases for iCoater, Reagent 1, and Reagent 2 were 12.72-, 16.81-, and 13.10-fold, respectively. Statistical analysis showed no significant differences in either the absolute cell numbers or the calculated proliferation rates between iCoater and the commercial reagents at both 4 h and 72 h (*p* > 0.05). Although primary duck liver cell populations inherently display cellular heterogeneity, the consistent attachment and growth kinetics observed across all replicates demonstrate the functional robustness of the iCoater. These biological properties were maintained even in samples stored at 4 °C for at least 12 months (Fig. 2c, Figure S2). Furthermore, cells cultured on the iCoater could be subcultured (Figure S3). Interestingly, an evaluation of the physical properties of this developed product revealed a pH of 4.9 – 5.4 and a low osmolality of 1 mOsm/L. These physicochemical properties are not optimal for cell culture. To address whether the autoclave sterilization alters the gelatin material, its molecular weight distribution and viscosity were evaluated with and without autoclave sterilization, where the non-autoclaved gelatin was dissolved at 60 °C (Figure S4 and Supplementary Table 1). The SDS-PAGE analysis showed a shift toward lower molecular weights after autoclave sterilization, while the measured viscosity values remained comparable. Although direct quantifications of surface adsorption amount and coating uniformity were not performed, the reproducible cell attachment and consistent growth kinetics across multiple independent replicates (Fig. 2 and Figures S2, S3, and S5) suggest that the iCoater provides a uniform and functional coating layer for practical applications. Although further characterization is required to evaluate molecular state shifts during extended storage, the iCoater successfully supported cell attachment and proliferation even after 12 months of storage. Removal of the iCoater after coating is a critical step in eliminating its physicochemical properties and ensuring an optimal environment for cell culture. As summarized in Supplementary Table 2, the pH and osmolality of the culture medium recovered after the iCoater coating process remained fully within the physiological range (pH 8.33 ± 0.01 (*n* = 3) and 287 ± 1 mOsmol/kg (*n* = 3), respectively), showing minimal deviations from the control (Supplementary Table 2). While conventional cell culture media are typically maintained around pH 7.2–7.4, the specific medium used in this study (10%YLP in I-MEM) inherently possesses a baseline pH of 8.11.

On Day 4, cell spreading and growth were clearly observed on iCoater-coated surfaces, whereas no growth was visible on non-coated surfaces (Fig. 2d, e). To evaluate the applicability of the platform, we quantitatively analyzed the cell adhesion, proliferation, and viability of C2C12 and HEK293T cells on the iCoater (Figure S5). Specifically, the iCoater coating significantly enhanced both initial attachment at 24 h and subsequent proliferation at 48 h for C2C12 cells. For HEK293T cells, while initial attachment at 24 h was comparable to the non-coated control, a significant increase in cell proliferation was observed at 48 h. These quantitative assays and subculture data demonstrated that the iCoater supported cell culture across these models, specifically for primary duck liver cells, C2C12 cells, and HEK293T cells. Future investigations incorporating a wider range of cell types, along with the evaluation of phenotype maintenance and long-term passaging, are expected to further expand the scope of its applicability.

In the context of cellular agriculture, our developed coating system demonstrates a practical approach to utilizing the comprehensive library of alternative scaffold materials and formats (compiled in Table 3 by Wang et al [17]). Furthermore, the food-grade processing and terminal heat-sterilization framework established in this study provides a vital concept and a manufacturing mindset for the future large-scale production and commercial processing of emerging scaffolding technologies [29].

Overall, the simple food production process established in this study clearly demonstrates that high sterility and long-term stability can be achieved without sacrificing any biological properties that are important for culture agents. For successful commercialization, evaluating the chronological consistency of not only biological performance but also physicochemical properties is a critical requirement. Therefore, monitoring detailed stability parameters, such as viscosity or molecular weight distribution over these storage periods, remains an essential point to be addressed in practical quality control designs.

### 3.2 Manufacturing robustness against water quality

To evaluate the industrial applicability of our process, we prepared iCoater using various water sources, ranging from soft water (hardness < 1.0 mg/L) to extremely hard water (1468 mg/L) (Table 1). As shown in Table 1, the terminal sterilization process successfully ensured sterility. Furthermore, analytical results confirmed that endotoxin levels remained below the detection limit (< 0.01 EU/mL) across all water sources, indicating that potable-quality water can be used directly without causing pyrogen contamination. No mycoplasma or bacterial growth was detected. The robustness of this technology is demonstrated by its compatibility with a wide range of water qualities, from soft water (below 60 mg/L) to very hard water (exceeding 300 mg/L), as defined in the World Health Organization (WHO) Guidelines for Drinking-water Quality [30]. These results indicate that the iCoater maintains its sterility and cell adhesion performance within the tested water hardness range.

### 3.3 Regulatory pathway: a realistic proposal

**Fig. 3:**
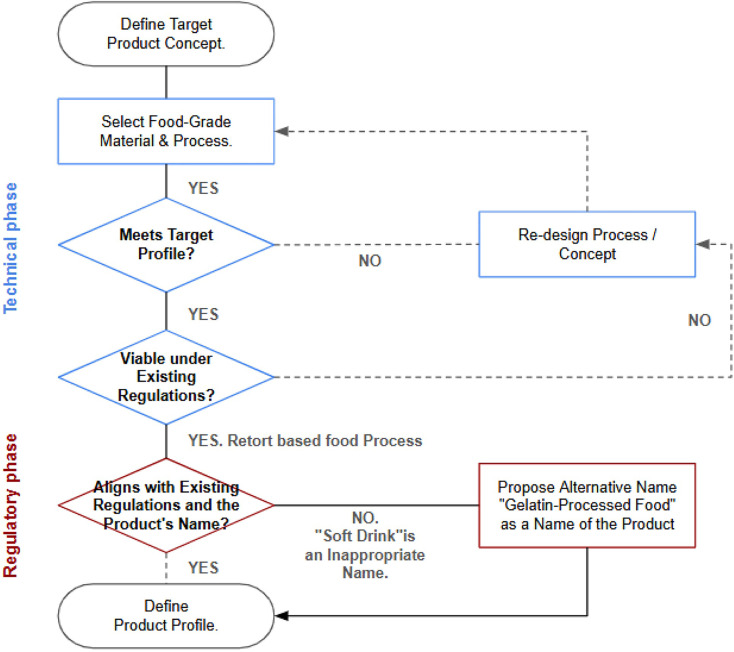
**Decision-making framework for developing food-grade cell culture reagents with iCoater as a case study.** Blue and red elements denote technical and regulatory decisions, respectively. The solid line indicates the pathway taken for iCoater, whereas dashed lines represent alternative routes not pursued.

Regarding the regulatory pathway, the key challenge was determining how to handle a product with dual functions as both a ‘food’ and a ‘culture agent’. We considered a realistic path to market launch within the interpretation of the existing regulations. First, iCoater belongs to a new category: ‘food-grade culture agents produced through food processing’. Therefore, to address this issue, we discussed the classification of this product as a food product based on the interpretation of Japan's Food Labeling Act (Article 5 of Act No 70 on Food Labeling (2013)) and the related Food Sanitation Act (Article 55 of Act No 233 on Food Sanitation (1947)) in consultation with multiple food regulatory experts and government officials (Supplementary Table 3). We focused on the relationship between the ‘business license’ of the manufacturing facility and the ‘name’ of the final product. A business license under the Food Sanitation Act indicates that the manufacturing facility and process meet the standards for safe production of a specific notification category (e.g., ‘soft drink manufacturing industry’) [31]. However, the purpose of product names under the Food Labeling Act is to convey the actual product to consumers accurately. These two do not necessarily have to be the same, and, for example, it is possible for a facility licensed as a ‘soft drink manufacturer’ to manufacture ‘consommé soup (prepared dish)’ using the same process. Thus, business licenses and food labeling are designed separately under the system, and the requirements that they must meet must be reconciled. Because the manufacturing process of this developed product is within the scope of the license for the ‘soft drink manufacturing industry’, it was technically possible to label it as ‘soft drink’. However, given its functionality, which is not intended for ‘drinking’, this labeling would deviate from the actual product and potentially undermine the integrity of the regulatory system. Therefore, we concluded that labeling it as ‘gelatin-processed food’ based on its main ingredient would be a more realistic option, as it would honestly represent the product's nature and prevent consumer misunderstanding (Fig. 3, Supplementary Table 4).

This issue of the discrepancy between ‘manufacturing approval’ and "product labeling" is not limited to Japan. The consistency of manufacturing permits and labeling systems may be a common issue globally, and certain commonalities can be observed in system structures. In the US, apart from the FDA's extensive ‘facility registration,’ the USDA asks whether a product's ‘Statement of Identity’ matches its actual state (“ [32]), which, like Japan's Food Labeling Act, has essentially the same structure in that problems arise when the ‘manufacturing approval’ and the ‘essence of the product’ do not match. In this context, a name such as ‘Soft Drink’ would be similarly unacceptable under USDA regulations. A descriptive name based on its intended use, such as ‘A gelatin-based solution as a processing aid,’ would be a more reasonable and practical option in the U.S. to clearly define its technical identity and prevent consumer confusion. These examples underscore that, although international standards such as Codex provide a foundation, navigating the regulatory landscape for such novel products ultimately requires individual discussions with each regulatory authority, as the administrative requirements are highly diverse and vary widely depending on the country [33].

Furthermore, in the European Union, where the classification of 'substances used as processing aids' is strictly governed under Regulation (EC) No 1333/2008, the integration of iCoater would require a clear distinction from food additives. The logical framework presented in Figure 3—aligning technical identity with functional labeling—provides a versatile methodology that can be adapted not only to Japan and the U.S. but also to the complex pre-market authorization processes of the EU's Novel Food regulations.

### 3.4 Limitations and future perspectives

The limitations of heat resistance highlight the challenges for future practical applications. For example, basal culture media, which are essential for cell growth, contain many heat-sensitive vitamins and amino acids, making this efficient heat sterilization process difficult to apply. Similarly, many functional components essential for cell growth, such as protein-based growth factors, are heat-labile, precluding the use of this method [19]. Therefore, a key point in future material development for cellular agriculture will be strategically selecting the optimal manufacturing process for each material's function. Materials such as heat-sensitive basal culture media require expensive sterile filtration processes. However, as shown in this study, selecting a highly heat-resistant coating agent such as gelatin offers options such as those shown in Fig. 1, potentially enabling practical applications within existing legal frameworks. In this sense, the use of porcine gelatin can be considered a realistic "bridging technology" that balances current practicality.

To demonstrate the versatility of this approach, we performed similar experiments using fish gelatin, a non-porcine ingredient. The results confirmed that the coating agent prepared by this process exhibited adhesion and proliferation properties comparable to those of primary cells, even when fish gelatin was used (Figure S6). This suggests that the heat sterilization process established in this study is not limited to specific ingredients but may also be applied to a variety of other heat-resistant food-grade ingredients. This versatility is crucial for addressing cultural or religious requirements, such as the need for Halal-compliant materials. Our finding that fish gelatin is an effective alternative demonstrates a viable path for developing products with broader social acceptance.

In the next stage, it will be important to explore non-animal-derived heat-stable ingredients that are compatible with this efficient manufacturing process. However, there are more important considerations than technical challenges. Adopting new ingredients also means facing new food-labeling challenges. For example, microbially derived cationic polylysine is a promising technological candidate [34]. It is currently approved as a food additive for use as a ‘preservative,’ and thus it may be possible to utilize this framework (labeling name: ‘Preservative (polylysine)’ or ‘Polylysine Preparation’). However, if a new, more efficient adhesion molecule produced by precision fermentation is developed, determining where it should be placed within the existing food classifications and how it should be labeled would be extremely difficult. Thus, the development of materials for cellular agriculture is always a combination of technological developments and regulatory science, and the consideration of both perspectives is essential.

Furthermore, a significant technical hurdle exists in the different mechanisms of action of these alternatives. For instance, polylysine promotes cell adhesion primarily through nonspecific electrostatic interactions between its cationic polymer chain and the negatively charged cell surface. This is in contrast to the mechanism of action of gelatin, which relies on specific binding between Arg-Gly-Asp motifs and integrin receptors. This distinction is critical because the choice of the coating agent must be compatible with the biological requirements of the target cell type, making the selection of alternatives a complex balance between cost, stability, and biological functionality.

Although this study focused on primary duck liver-derived cells, the well-established RGD-mediated mechanism suggests potential efficacy for a wide range of avian and mammalian cells. Future studies should not only validate this versatility across different species but also incorporate molecular assessments, such as quantifying the expression of cell adhesion molecules, to provide a ligand-level understanding of the coating performance.

As a roadmap for future practical implementation, several industrial and regulatory steps remain to be established. These include transitioning from laboratory-scale experiments to industrial-scale validation involving initial bioburden assessments and package integrity testing. Additionally, although basic consistency was confirmed at the laboratory scale, we acknowledge that high-temperature heat treatment may alter the molecular weight distribution, aggregation state, and surface adsorption capacity of gelatin, necessitating more rigorous quality characterization during scale-up. Furthermore, future investigations will need to encompass cell culture evaluation under antibiotic-free conditions and detailed quantitative cost modeling for scaled production. Finally, evaluating potential coating residues on the harvested cells, performing comprehensive food-safety assessments for formal regulatory approval, and validating product stability under varying packaging, handling, and transport conditions represent necessary milestones for subsequent development.

## 4 Conclusions

We successfully developed a practical and robust coating agent by combining food-grade gelatin with terminal sterilization, which is a common food manufacturing process. Concurrently, we proposed a viable regulatory pathway for this novel product category by classifying it as a "gelatin-processed food" under existing regulatory frameworks. This integrated strategy provides a crucial blueprint for developing compliant agents, thereby accelerating the industrialization of cellular agriculture.

## CRediT authorship contribution statement

**Hiroaki Hatano:** Writing – review & editing, Writing – original draft, Supervision, Project administration, Investigation, Formal analysis, Data curation, Conceptualization. **Misaki Sawada:** Visualization, Validation, Resources, Methodology, Investigation, Formal analysis, Data curation. **Naomi Nakamura:** Validation, Formal analysis, Data curation. **Misaki Kitsukawa:** Data curation. **Azusa Hayakawa:** Data curation. **Hiroaki Kondo:** Supervision. **Ikko Kawashima:** Supervision, Funding acquisition.

## Declaration of competing interest

The authors declare the following financial interests/personal relationships which may be considered as potential competing interests: All authors are employees of IntegriCulture Inc. IntegriCulture Inc. has developed and intends to commercialize the food-grade coating technology (iCoater) described in this manuscript. This work was supported by IntegriCulture Inc.

## Data Availability

Data will be made available on request.

## Appendix Supplementary materials

Image, application 1

Image, application 2

Image, application 3

Image, application 4

Image, application 5

Image, application 6

Image, application 7

Image, application 8

Image, application 9

Image, application 10
