# Supplementary material for: Terminal sterilization of food-grade coatings for cellular agriculture: robustness against water quality and regulatory feasibility
Source: Biotechnol Rep (Amst). 2026 Aug 4;51:e00976. doi: 10.1016/j.btre.2026.e00976 (PMC13486997; doi:10.1016/j.btre.2026.e00976)
Supplement: Supplementary file 1 [file mmc1.docx]

**Figure S1. Validation of sterilization efficiency via a spore challenge test using biological indicators**. The experimental setup and colorimetric results after a 48-h incubation at 56°C are shown. (Left) Visual evaluation of the 3M Attest 1262P biological indicators. Vials ① to ④ remained negative (N, purple), indicating the complete inactivation of Geobacillus stearothermophilus spores under the retort thermal sterilization process (121°C for 20 min). The non-autoclaved control (N.C.) turned positive (P, yellow), confirming spore viability and proper incubation conditions. (Right) Schematic diagram illustrates the spatial distribution of the biological indicators inside the autoclave chamber during the process:

①: Top shelf, inside the 2 L container (liquid phase).
②: Top shelf, outside the container (chamber environment).
③: Bottom shelf, inside the 2 L container (liquid phase).
④: Bottom shelf, outside the container (chamber environment).
