# Supplementary material for: Terminal sterilization of food-grade coatings for cellular agriculture: robustness against water quality and regulatory feasibility
Source: Biotechnol Rep (Amst). 2026 Aug 4;51:e00976. doi: 10.1016/j.btre.2026.e00976 (PMC13486997; doi:10.1016/j.btre.2026.e00976)
Supplement: Supplementary file 2 [file mmc10.docx]

**Supplementary Table 4. Proposed food label design for iCoater as a "Gelatin Processed Food.”**

| Name | Gelatin-Processed Food |
| --- | --- |
| Ingredients | Gelatin (porcine-derived) |
| Contents | 500 mL |
| Best Before | 12 months from the manufacturing date |
| Storage | Store at room temperature away from direct sunlight. |
| Allergen Information | Contains: Gelatin |
