# Supplementary material for: Terminal sterilization of food-grade coatings for cellular agriculture: robustness against water quality and regulatory feasibility
Source: Biotechnol Rep (Amst). 2026 Aug 4;51:e00976. doi: 10.1016/j.btre.2026.e00976 (PMC13486997; doi:10.1016/j.btre.2026.e00976)
Supplement: Supplementary file 3 [file mmc2.docx]

**Figure S2. Long-term stability of iCoater. Primary duck liver-derived cells were cultured for 1 day and 3 day (72 h) on plates coated with either freshly prepared iCoater (iCoater fresh: within 1 week) or iCoater that had been stored at 4°C for 9 months**. Data are presented as mean ± SD (*n*=3: ** *p* < 0.01) and ns (not significant).
