# Supplementary material for: Terminal sterilization of food-grade coatings for cellular agriculture: robustness against water quality and regulatory feasibility
Source: Biotechnol Rep (Amst). 2026 Aug 4;51:e00976. doi: 10.1016/j.btre.2026.e00976 (PMC13486997; doi:10.1016/j.btre.2026.e00976)
Supplement: Supplementary file 4 [file mmc3.docx]

**Figure S3. The developed coating agent supports subsequent passaging of primary cells.** Primary duck liver-derived cells were cultured on iCoater for 3-4 days. The cells were then detached (passaged), reseeded onto new plates, and coated with iCoater. The bright-field images show proliferating cells before they are detached. Scale bar = 400 µm.
