# Supplementary material for: Terminal sterilization of food-grade coatings for cellular agriculture: robustness against water quality and regulatory feasibility
Source: Biotechnol Rep (Amst). 2026 Aug 4;51:e00976. doi: 10.1016/j.btre.2026.e00976 (PMC13486997; doi:10.1016/j.btre.2026.e00976)
Supplement: Supplementary file 5 [file mmc4.docx]

**Figure S4. SDS-PAGE analysis of gelatin molecular weight distribution showing a decrease in molecular weight by autoclave sterilization.** Lanes represent the molecular weight marker (kDa), autoclaved gelatin (iCoater), and non-autoclaved gelatin (dissolved at 60°C). Autoclave sterilization induces a shift toward lower molecular weights compared to the non-autoclaved gelatin.
