# Supplementary material for: Terminal sterilization of food-grade coatings for cellular agriculture: robustness against water quality and regulatory feasibility
Source: Biotechnol Rep (Amst). 2026 Aug 4;51:e00976. doi: 10.1016/j.btre.2026.e00976 (PMC13486997; doi:10.1016/j.btre.2026.e00976)
Supplement: Supplementary file 6 [file mmc5.docx]

**Figure S5. Evaluation of cell viability and proliferation on non-coated and iCoater-coated surfaces.** Luminescence intensity was measured at 24 h and 48 h post-seeding to evaluate the proliferation of (A) C2C12 cells and (B) HEK293T cells on non-coated (No Coat) and iCoater-coated surfaces (iCoater coat). Data is represented as mean ± standard deviation (*n* = 6). Statistical significance determined by t-test is indicated as *p* < 0.01 and ns (not significant).
