# Supplementary material for: Terminal sterilization of food-grade coatings for cellular agriculture: robustness against water quality and regulatory feasibility
Source: Biotechnol Rep (Amst). 2026 Aug 4;51:e00976. doi: 10.1016/j.btre.2026.e00976 (PMC13486997; doi:10.1016/j.btre.2026.e00976)
Supplement: Supplementary file 7 [file mmc6.docx]

**Figure S6. Applicability of the terminal sterilization process to fish-derived gelatin. Primary duck liver-derived cells were cultured on iCoater for 3-5 days.** The bright-field image shows proliferating cells before detachment. Scale bar = 400 µm.
