# Supplementary material for: Terminal sterilization of food-grade coatings for cellular agriculture: robustness against water quality and regulatory feasibility
Source: Biotechnol Rep (Amst). 2026 Aug 4;51:e00976. doi: 10.1016/j.btre.2026.e00976 (PMC13486997; doi:10.1016/j.btre.2026.e00976)
Supplement: Supplementary file 8 [file mmc7.docx]

**Supplementary Table 1.**  Viscosity of coating solutions before and after the manufacturing process.

| Sample / Condition | Viscosity (mPa·S) |
| --- | --- |
| iCoater (autoclaved) | 1.6 |
| Non-autoclaved gelatin (dissolved at 60°C) | 2.0 |

Note:
Viscosity measurements were performed as a contract analytical service by the Japan Food Research Laboratories (Tokyo, Japan). Samples were analyzed at a controlled temperature of 5°C using a Brookfield rotational viscometer (Type B, Model TVB-15; Toki Sangyo Co., Ltd., Tokyo, Japan) equipped with an ML adapter at a rotor speed of 60 r/min.
