# Supplementary material for: Terminal sterilization of food-grade coatings for cellular agriculture: robustness against water quality and regulatory feasibility
Source: Biotechnol Rep (Amst). 2026 Aug 4;51:e00976. doi: 10.1016/j.btre.2026.e00976 (PMC13486997; doi:10.1016/j.btre.2026.e00976)
Supplement: Supplementary file 9 [file mmc8.docx]

**Supplementary Table 2.**  Effect of iCoater-coating process on pH and osmolality of the culture medium.

| Sample / Condition | pH | Osmolality (mOsmol/kg) |
| --- | --- | --- |
| Control (Fresh medium) | 8.11 | 298 ± 0 |
| iCoater-treated medium (*n* = 3) | 8.33 ± 0.01 | 287 ± 1 |
| iCoater stock solution | 5.48 | 0 ± 1 |

Note:
Data are presented as the mean ± SD. The baseline pH of the fresh culture medium (10%YLP in I-MEM) used in this study is inherently 8.11. For the iCoater-treated medium, fresh culture medium was introduced into T175 flasks after the standard iCoater decanting procedure, agitated, and recovered for analysis (*n* = 3 independent replicates).
