# Supplementary material for: Terminal sterilization of food-grade coatings for cellular agriculture: robustness against water quality and regulatory feasibility
Source: Biotechnol Rep (Amst). 2026 Aug 4;51:e00976. doi: 10.1016/j.btre.2026.e00976 (PMC13486997; doi:10.1016/j.btre.2026.e00976)
Supplement: Supplementary file 10 [file mmc9.docx]

**Supplementary Table 3. Comparative Analysis of Two Potential Product Names for iCoater under Japanese Food Regulations.**

|  | **Option 1: Name as "Soft Drink"** | **Option 2: Name as "Gelatin-Processed Food”** |
| --- | --- | --- |
| Primary Justification | Aligns formally with the manufacturing license ("Soft Drink Manufacturing") of the facility. | Aligns with the product's actual nature and composition (made from gelatin). |
| Governing Principle | Based on the manufacturing process being identical to that of soft drinks. | Based on the principle of accurately communicating the product's identity to prevent consumer misunderstanding. |
| Risk of Consumer Misunderstanding | High. - Consumers may mistakenly expect it to be a beverage. | Low. - The name clearly indicates the primary ingredient and that it is a processed food, not a drink. |
| Regulatory Challenge | The name does not reflect the product's true function (cell coating), which contradicts the spirit of the Food Labeling Act. | Although rational, it requires establishing a new understanding of a "food" that is also a "cell culture reagent". |
| Manufacturing License | The product is manufactured under a "Soft Drink Manufacturing" license. | The product is also manufactured under a "Soft Drink Manufacturing" license, as the process is identical. |
